# Implementation of initiatives designed to improve healthcare worker health and wellbeing during the COVID-19 pandemic: comparative case studies from 13 healthcare provider organisations globally

**DOI:** 10.1186/s12992-022-00818-4

**Published:** 2022-02-22

**Authors:** N O’Brien, K Flott, O Bray, A Shaw, M Durkin

**Affiliations:** Institute of Global Health Innovation, Imperial College London, London, United Kingdom

**Keywords:** Global institutions/organizations, Human resources for health, Healthcare workers, Health care planning, COVID-19, Outbreaks, Communicable disease, Psychosocial impacts

## Abstract

**Background:**

Healthcare workers are at a disproportionate risk of contracting COVID-19. The physical and mental repercussions of such risk have an impact on the wellbeing of healthcare workers around the world. Healthcare workers are the foundation of all well-functioning health systems capable of responding to the ongoing pandemic; initiatives to address and reduce such risk are critical. Since the onset of the pandemic healthcare organizations have embarked on the implementation of a range of initiatives designed to improve healthcare worker health and wellbeing.

**Methods:**

Through a qualitative collective case study approach where participants responded to a longform survey, the facilitators, and barriers to implementing such initiatives were explored, offering global insights into the challenges faced at the organizational level. 13 healthcare organizations were surveyed across 13 countries. Of these 13 participants, 5 subsequently provided missing information through longform interviews or written clarifications.

**Results:**

13 case studies were received from healthcare provider organizations. Mental health initiatives were the most commonly described health and wellbeing initiatives among respondents. Physical health and health and safety focused initiatives, such as the adaption of workspaces, were also described. Strong institutional level direction, including engaged leadership, and the input, feedback, and engagement of frontline staff were the two main facilitators in implementing initiatives. The most common barrier was HCWs’ fear of contracting COVID-19 / fear of passing COVID-19 to family members. In organizations who discussed infection prevention and control initiatives, inadequate personal protective equipment and supply chain disruption were highlighted by respondents.

**Conclusions:**

Common themes emerge globally in exploring the enablers and barriers to implementing initiatives to improve healthcare workers health and wellbeing through the COVID-19 pandemic. Consideration of the themes outlined in the paper by healthcare organizations could help influence the design and deployment of future initiatives ahead of implementation.

## Background

Severe acute respiratory syndrome coronavirus (SARS-CoV-2), henceforth described as COVID-19, was first identified in Wuhan, China in December 2019 and has since spread to more than 200 countries [1]. Healthcare workers (HCWs) at the frontline of the COVID-19 pandemic are at a disproportionate risk of adverse physical and psychological outcomes [2]. The true scale of COVID-19’s impact on health and wellbeing is not yet known, however Amnesty International found that at least 17,000 healthcare workers around the world died in the first year of the pandemic, a substantial increase from more than 3,000 deaths reported in research published in July 2020 [3, 4]. Data from surveys around the world administered during the COVID-19 pandemic, as well as other pandemics and epidemics, also found that HCWs experienced concerns about their own health and fear of transmitting the virus to family, as well as increased levels of depression, anxiety, distress and insomnia [5–8]. Nurses, female workers, frontline workers, younger medical staff, and workers in areas with higher infection rates have been identified as the groups most likely to suffer severe adverse psychological outcomes [7].

Protecting HCWs requires a comprehensive approach to address multiple aspects of health and wellbeing. Healthcare facilities must develop infection prevention and control as part of protecting physical health and wellbeing, engineering changes to workflow and administrative systems [5]. Infection prevention and control (IPC) are measures or initiatives that aim to protect healthcare workers, patients and visitors from aquiring an infection in a healthcare organization, and to control infection transmission when identified. Examples include the provision and use of personal protective equipment (PPE), safe injection practices, and the promotion of hand hygiene. However, such initiatives are not necessarily simple to implement given financial and human resource constraints, among other challenges. Notably, many countries have struggled to secure PPE for their health workers, partly because of shortages on the international market [5]. However, there are also instances of corruption and misuse of funds, including for contracts for the procurement of PPE [5].

Initiatives to support physical health must be underpinned by strong leadership and appropriate psychological support for staff [9]. Mental health support services are services or initiatives that aim to support the mental health of healthcare workers. Workplace initiatives can improve the working lives of HCWs as well as mental wellbeing [2]. Explicit support services designed to support mental health can include a staff support telephone hotline, the availability of wellbeing resources such as apps or mindfulness videos, and a peer to peer listening service. Non explicit support services are services or initiatives set up without mental health support as the primary goal, but do have a positive impact on mental health. Examples include the provision and use of PPE, which can reduce HCWs concerns over their health and spreading infections to their families [2]. Non-explicit initiatives may seek to ease caregiver or childcare burden or lessen financial stressors, such as hazard pay, for example, to mitigate negative mental health outcomes [2].

The importance of HCWs to a well-functioning health system is not always acknowledged or backed up with appropriate responses from systems or leaders. Developing and launching initiatives designed to address and reduce health and wellbeing challenges whilst under time, human resource and financial pressures is a key challenge for many health systems and institutions globally. Our study addresses a research gap by unpacking the facilitators and barriers to the implementation of initiatives to improve the health and wellbeing of HCWs through the COVID-19 pandemic. While initiatives have been rolled out globally, the health and wellbeing of HCWs continues to be a major concern in healthcare organizations around the world and so we need to better understand how best to support them [10, 11]. This paper presents a series of findings on the facilitators and barriers to implementing health and wellbeing initiatives, based on case studies from health systems globally, to inform and generate transferable lessons and facilitate shared learning.

## Methodology

### Design and Theoretical Approach

A collective case study approach was selected as the research method as it allows in-depth, multi-faceted explorations of complex issues in their real-life settings [12]. The case study approach is an established research design and is sometimes referred to as a "naturalistic" design as it explores an event or phenomenon in depth and in its natural context. This contrasts with an "experimental" design, where investigators seek to exert control over and manipulate the variable(s) of interest[12]. The *collective case study* involves studying multiple cases simultaneously to generate a broader appreciation of a particular issue [12]. Gilson et al. (2011) note that in studies with multiple cases, systematic and deliberate cross-case comparison supports analytic generalization, not to draw conclusions that can be statistically generalized to a wider study population, or that will hold across time and place, but rather towards “general conclusions that, although derived from a limited number of particular experiences, provide theoretical insights that can be put forward for consideration, and testing, in other, similar situations [13].

The research was grounded in implementation research, which refers to “the application of effective and evidence‐based interventions, in targeted settings, to improve the health and well‐being of specific population groups” [14]. Within implementation research, “implementation science” describes the scientific study of methods that take findings into practice, while “effective implementation” refers to the process whereby an intervention is appropriately and successfully executed [15]. Considering initiatives to improve HCWs health and wellbeing during the COVID-19 pandemic through the lens of implementation research encourages questions to be asked about whether, and if so how, initiatives can make a difference to HCWs and patients. Questions are also raised about the practice of a healthcare delivery team, and whether bringing new knowledge into one setting automatically, or with effort, enables its applicability in another. Answers to such questions will encourage better, more targeted service provision and policy development, closely linking HCWs health and wellbeing and the delivery of healthcare in a pandemic situation with rigorous evidence.

### Methods

#### Data collection and facility/participant selection

The research participants comprised of representatives from 13 healthcare provider organizations from 13 countries. The selection of participants was done through the following criteria: individuals who have oversight of the management of healthcare provision within a healthcare institution and have permission from the relevant institution to share information about initiatives developed/implemented for healthcare workers in response to the COVID-19 pandemic. The identification and recruitment of participants was initiated through the Imperial College London Leading Health Systems Network (LHSN), the NIHR Imperial Patient Safety Translational Research Centre (PSTRC), and through the networks of the research team. The research team initially approached 20 contacts based on their assessment of their existing contacts in healthcare organisations around the world. The assessment process towards contact selection focused on identifying contacts to approach that were 1) geographically diverse to facilitate international comparisons between health systems (e.g. equal numbers where possible from Africa, East Asia and Pacific, Europe and Central Asia, Latin America and the Caribbean, Middle East and North Africa, and South Asia), 2) at the healthcare provider level to examine local level decision making, and 3) from diverse healthcare provider organizations to examine differences between types of provider (e.g. public, private, faith-based, parastatal). The research team provided potential participants information on the aims of the research and the study protocol before informed consent was obtained from those who agreed to take part (*N *= 13). The case studies were collected via a survey developed in Qualtrics. The questions were developed and tested internally by the research team. Questions were focused on recently implemented initiatives and facilitators and barriers to their implementation, offering participants the opportunity to write free text responses. Specific follow up questions were sent via email to each of the participants and online calls were held on Microsoft Teams where required. Questions asked during the calls focused on clarifying the responses to the initial survey. Ethical approval was provided by the Imperial College Research Ethics Committee (ICREC reference: 20IC6277). The research was conducted online between 22nd September 2020 and 22nd December 2020.

### Data analysis

The NVivo 1.0 (QSR International) qualitative data analysis computer software package was used to systematically code the data and assist analysis, especially in cataloguing codes to develop and connect codes into wider themes. The research team used a “ground up” approach, developing codes derived from the primary data, and linked concepts and codes to specific themes. The four theme nodes that formed the starting point of the analysis were: initiatives, facilitators, barriers, and lessons learned. NO (author 1) and OB (author 2) independently coded the data and met to review and address discrepancies. During the meeting to review discrepancies, each author (1 and 2) presented their justification for coding the data in question and subsequently discussed and came to agreement on the codes most appropriate for data with discrepancies. AS (author 3) reviewed the final analysis to enhance internal validity, focusing particularly on the final coding of discrepancies by authors 1 and 2. Finally, as part of the analysis process, ‘word frequency queries’ were run on NVivo to identify words that occurred most often in the dataset, as well as their relative and absolute frequency to determine the most mentioned aspects of the research topic.

## Results

**Fig. 1:**
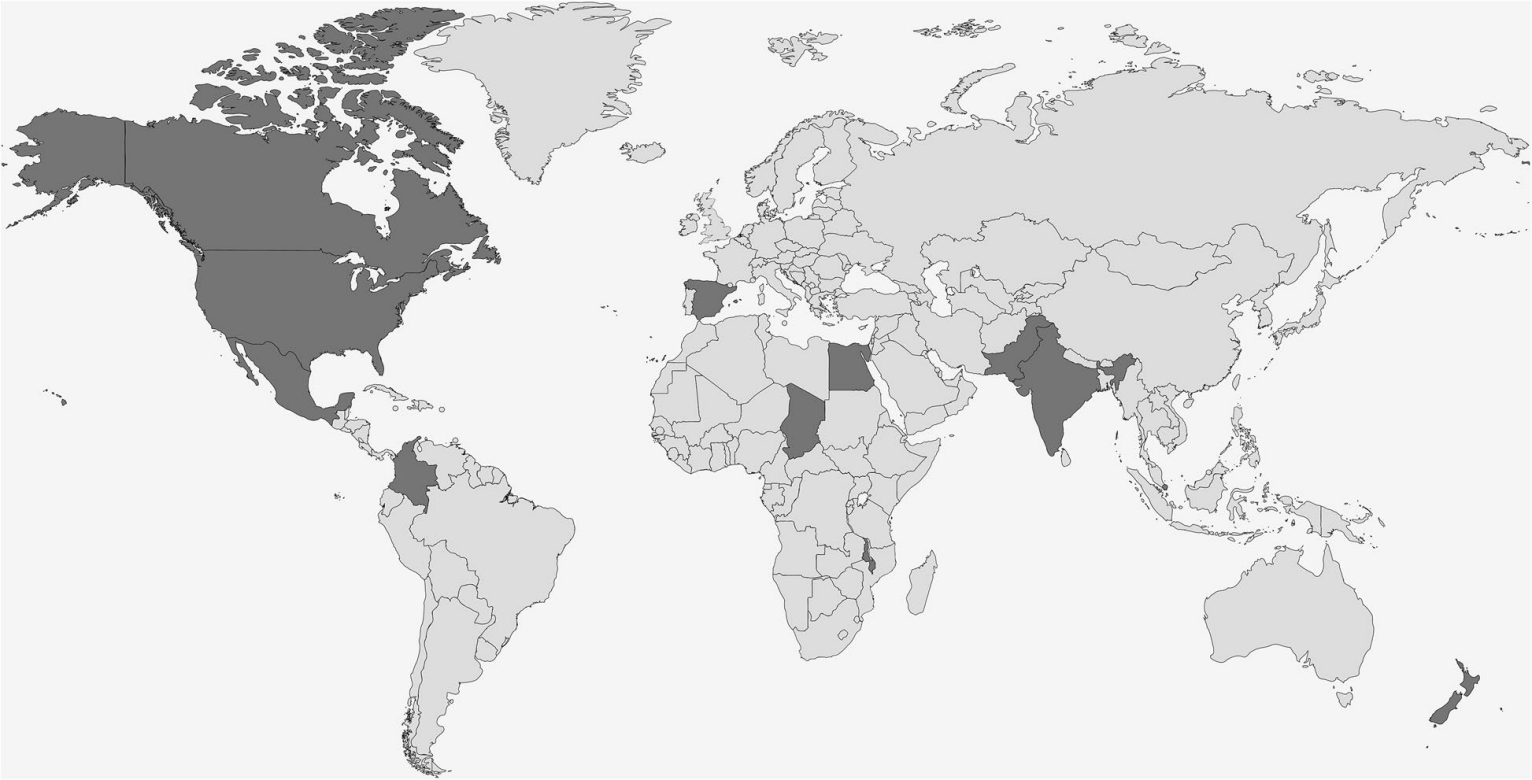
Participants by country

We received a range of responses from 13 participant organizations, outlining one or several initiatives at the facility level. In three cases responses focused on initiatives at the systems level from the perspective of a World Health Organization (WHO) Regional Office, a national ministry of health and a national patient safety institute. Of the remaining local healthcare organizations, 6 were public sector institutions and 4 were private sector. The participant countries are outlined in Fig. 1. Table 1 outlines a summary of the national

**Table 1:** Details of the COVID-19 pandemic in participant countries

**Country**	**Summary of health system**	**Date of first case (2020)**	**Estimated total cumulative COVID-19 Cases per 1 million population**^**a**^[16]	**Estimated total culmative COVID-19 Deaths per 1 million population**^**a**^[16]
Canada	Decentralized, universal, publically funded health system [17].	26^th^ January [18]	4,310	249
Chad	Mix of severely limited public and private healthcare providers [19].	19^th^ March [20]	74	5
Colombia	Mix of parallel public and private insurers and healthcare providers [21].	6^th^ March 30]	16,539	519
Egypt	Mix of public, parastatal and private insurers and healthcare providers [22].	13^th^ February [23]	1,012	58
India	Mixed financing system, with decentralized, universal, publically funded health system and private sector [24].	30^th^ January [18]	4,746	74
Kenya	Mix of public and private, for-profit and nonprofit, and faith-based healthcare providers [25, 26].	13^th^ March [27]	724	13
Malawi	Mix of public and private, for-profit and nonprofit, and faith-based healthcare provider [28].	2^nd^ April [29]	302	9
Mexico	Mixed financing system, with employment-based social insurance schemes, public system for the uninsured, and a private sector [30].	28^th^ February [18]	5,814	609
New Zealand	Universal, publically funded health system, delivery system regionally administered [31].	28^th^ February [32]	311	5
Pakistan	Mix of parallel public and private healthcare providers [33].	26^th^ February [34]	1,424	29
Singapore	Mixed financing system, with public statutory insurance system [35].	23^rd^ January [36]	9,880	5
Spain	Universal, publically funded health system, delivery system regionally administered [37].	1^st^ February [18]	16,895	686
United States of America	Mix of public and private, for-profit and nonprofit insurers and healthcare providers [38].	22^nd^ January [18]	21,922	626

^a^Figures on 4^th^ October 2020

health system, the date of the first reported case, and estimated total COVID-19 cases and deaths in each of the participant countries.

Table 2 outlines the types of initiatives reported at the country level, as well as the associated facilitators and barriers generalized across the initiatives in each country context.

**Table 2:** Types of initiatives implemented, and facilitators/barriers identified

**Country**	**Intervention(s) reported**	**Facilitators**	**Barriers**
Canada	Support programs for psychological and mental health.	Organizational readiness	Challenges in engaging staff on the uptake of initiatives Inadequate external knowledge translation / changing national guidelines
Chad	IPC surveillance, training, and PPE provision	Government/national engagement with the organization and/or intervention(s) Communication across the organization	HCWs fear of contracting COVID-19 / fear of passing COVID-19 to family members
Colombia	Health and safety at work initiatives, including adaptation of workplaces. IPC surveillance, training, and PPE provision.	Adequate financial resources Commitment from leadership Staff input, feedback, and engagement Teamwork across the organization	Lack of adequate education and training for staff / Misinformation HCWs fear of contracting COVID-19 / fear of passing COVID-19 to family members The wider political and public health context
Egypt	Active surveillance of psychological and mental health of staff. Health and safety at work initiatives, including adaptation of workplaces.	Commitment from leadership Organizational readiness Staff input, feedback, and engagement	HCWs fear of contracting COVID-19 / fear of passing COVID-19 to family members Lack of human resources within the organization
India	Health and safety at work initiatives, including adaptation of workplaces. IPC surveillance, training, and PPE provision. Support programs for psychological and mental health. Redeployment and workload re-distribution.	Commitment from leadership Communication across the organization Development of guidelines and protocols Government/national engagement with the organization and/or intervention(s)	Inadequate knowledge translation / changing guidelines HCWs fear of contracting COVID-19 / fear of passing COVID-19 to family members Lack of human resources within the organization
Kenya	Health and safety at work initiatives, including adaptation of workplaces. IPC surveillance, training, and PPE provision Support programs for psychological and mental health.	Adequate financial resources Government/national engagement with the organization and/or intervention(s)	PPE challenges
Malawi	IPC surveillance, training, and PPE provision Support programs for psychological and mental health. Recognition and awards for staff.	Staff input, feedback, and engagement	Challenges in engaging staff on the uptake of initiatives HCWs fear of contracting COVID-19 / fear of passing COVID-19 to family members PPE challenges The wider political and public health context
Mexico	Health and safety at work initiatives, including adaptation of workplaces. IPC surveillance, training, and PPE provision	Adequate financial resources Communication across the organization Staff input, feedback, and engagement	PPE challenges
New Zealand	Creation of new role for staff support	Commitment from leadership Communication across the organization Staff input, feedback, and engagement Teamwork across the organization The wider political and public health content	Challenges in engaging staff on the uptake of initiatives Staff exhaustion
Pakistan	Health and safety at work initiatives, including adaptation of workplaces. IPC training and PPE provision. Support programs for psychological and mental health.	Staff input, feedback, and engagement	
Singapore	Health and safety at work initiatives, including adaptation of workplaces. IPC surveillance, training, and PPE provision. Redeployment and workload re-distribution.	Commitment from leadership Communication across the organization Government/national engagement with the organization and/or intervention(s)	“Fake news” and misinformation circulating on social media PPE challenges
Spain	Support programs for psychological and mental health.	Pressure of the media to address HCWs health and wellbeing	Challenges in engaging staff on the uptake of initiatives Lack of focus on teams and organizations in developing initiatives
United States	IPC surveillance, training, and PPE provision Support programs for psychological and mental health. Recognition and awards for staff. Redeployment and workload re-distribution.	Commitment from leadership Effective infection prevention and control Staff input, feedback, and engagement	“Fake news” and misinformation circulating on social media Inadequate external knowledge translation / changing national guidelines Lack of human resources within the organization The wider political and public health context Trust in the organization or health system

Across the countries, initiatives focused on physical health, including infection prevention and control (IPC), and mental health. Mental health initiatives were most commonly described among the respondents, with various initiatives designed to combat mental health as a standalone concern or as part of a more holistic approach to health and wellbeing, such as the management of staff rota to balance increasing staffing demands while seeking to reduce burnout. Respondents noted that mental health initiatives developed by their institutions were designed to address burnout, compassion fatigue, stress, and trauma. Fear of infection, both individually and bringing COVID-19 home to family members, was commonly cited as a major driver of mental ill health among staff. Notably, fear as a barrier was exclusively mentioned by healthcare organisations in low- or middle-income countries (LMICs), perhaps due to the resource constraints (e.g. fewer human resources to treat patients, PPE and equipment challenges) more acutely found in LMICs. However, additional research is required to better understand the role of fear in different organizational, health system, and geographic contexts. Examples of standalone mental health initiatives highlighted by respondents included: peer-to-peer support programs, support hotlines and psychological first aid.

Physical health initiatives were captured via several different types of initiative described. Initiatives that adapted the workplace, such as actions towards health and safety compliance in the COVID-19 environment and actions to reduce the transmission risk to HCWs were commonly noted by respondents. The implementation of initiatives involving the use of PPE were highlighted by more than half of respondents as a key element of health and wellbeing addressed by the organization following the onset of the COVID-19 pandemic. PPE initiatives were often closely related to wider IPC and surveillance. Respondents across a range of geographic areas, types of health system, and public/private orgnaizations noted PPE challenges as a barrier to implementation of initiatives, highlighting the universality of this barrier during the study period. Similarly, training and awareness raising initiatives and guidance for staff were outlined in several subject areas, including IPC.

Initiatives focused on administration, management and adapted workplace, and health and safety largely overlapped with the physical and mental health initiatives to support the health and wellbeing of HCWs. For example, the set-up of “hot and cold” wards, wards for COVID-19 positive patients and wards without COVID-19 positive patients, with different rules and PPE requirements to reduce infection transmission among patients and staff was designed to reduce the risk of physical ill health among HCWs, but also reassure HCWs working in the wards that safety was a priority. Leadership engagement initiatives, including the introduction of COVID-19 information ward rounds and designated COVID-19 leadership liaisons were described commonly by respondents, as was the development of awards to recognize outstanding performance and to boost morale.

### Facilitators to implementation

Several facilitators of implementation were described by the respondents (see Table 3). The two main facilitators noted were staff input, feedback, and engagement (*N *= 7) and commitment from leadership *(N *= 6). Other common facilitators were communication across the organization (*N *= 5), government/national engagement with the organization and/or intervention(s) (*N *= 4) and adequate financial resources (*N *= 3). At the facility level, organizational readiness (*N *= 2), teamwork across the organization (*N *= 2), effective infection prevention and control (*N* = 1), and the development of guidelines and protocols (*N *= 1) were also considered enablers in implementing initiatives to improve HCWs health and wellbeing in the pandemic context. Respondents also noted pressure from the media to address HCWs health and wellbeing (*N *= 1), and the wider political and public health context (*N *= 1), such as low infection rates in the community, as external facilitators to implementation.

**Table 3:** Summary of facilitators and barriers to intervention implementation

**Facilitators**	**Barriers**
**Internal**	
Commitment from leadership	Engaging staff on the uptake of initiatives
Communication across the organization	HCWs’ fear of contracting COVID-19
Development of guidelines and protocols	Lack of adequate education and training for staff /
Effective infection prevention and control	Lack of focus on teams and organizations in developing initiatives
Organizational readiness	Lack of human resources within the organization
Staff input, feedback, and engagement	Staff exhaustion
Teamwork across the organization	
**External**	
Government/national engagement with the organization and/or intervention(s)	The wider political and public health context
Pressure from the media to address HCWs health and wellbeing	
The wider political and public health context	
**Internal and external**	
Adequate financial resources	Inadequate knowledge translation / changing guidelines
	Fear of passing COVID-19 to family members
	“Fake news” and misinformation circulating on social media
	PPE challenges
	Lack of trust in the organization or health system

### Barriers to implementation

Several barriers to implementation were described by the respondents (see Table 3). The most noted barrier was HCWs’ fear of contracting COVID-19 / fear of passing COVID-19 to family members (*N *= 5). Similarly, respondents commonly noted challenges in engaging staff on the uptake of initiatives, largely due to exhaustion and burnout, stigma around the need to utilize initiatives, or cynicism on the value of utilizing initiatives (*N *= 4), PPE challenges (*N *= 4), a lack of human resources (*N *= 3), inadequate external knowledge translation / changing national guidelines (*N *= 3), and the wider political and public health context, such as political priorities, the balance between economic prosperity and public health decision-making, and non-COVID-19 clinical demands and priorities (*N *= 3). At the facility level, lack of adequate education and training for staff / misinformation (*N *= 1), exhaustion (*N *= 1), and a lack of focus on teams and organizations in developing initiatives (*N *= 1) were also considered barriers in implementing initiatives to improve the health and wellbeing of HCWs in the pandemic context. Respondents also noted “Fake news” and misinformation circulating on social media (*N *= 2), and lack of trust in the organization or health system (*N *= 1) as barriers to implementation that are both internal and external to the organisation.

## Discussion

### The role of leadership and effective engagement in multi-level coordination

Based on the facilitators highlighted by participants, including staff input, feedback and engagement, the role of leadership, organizational readiness, the development of guidelines and protocols, and teamwork across the organization, it is clear that multi-level coordination can act as a facilitator of initiatives. Multi-level coordination and preparedness, which we define as the range of actions undertaken simultaneously and with input from a range of stakeholders that are required to prepare the organization for a pandemic situation, facilitates frontline healthcare providers in developing, rolling out and managing initiatives to improve the health and wellbeing of staff.

Effective coordination within organizations, as well as with external partners, regional and national government, and in line with guidance from the World Health Organization, is a critical element of managing HCWs health and wellbeing during a pandemic situation. As developing and maintaining good multi-level coordination is a complex and challenging task, when organizations are confronted with a range of competing priorities, the importance of forward planning for a pandemic situation is critical. Human and financial resources should be made available to organizations to work towards this goal. Policies and guidelines should be in place to ensure both mental and physical safety of HCWs before a pandemic and updated based on emerging local and international guidance following the onset of the pandemic.

The most published coordination challenges through the COVID-19 outbreak thus far have focused on the provision of personal protective equipment (PPE) and guidance on how it should be used by HCWs. A variety of challenges have been outlined in the literature [9, 39, 40], as well as by respondents of the study, covering procurement, including price regulation and shortages, PPE quality, distribution, provision, and guidelines on use. One respondent summed up the multi-level challenges.

“It was unclear if supply chains of medical equipment (including PPE) would be disrupted. This potential threat to [organization’s] supply of equipment was compounded by early national epidemic curve projections predicting a surge in COVID-19 admissions to hospitals, which would have driven up healthcare demand and use of medical equipment. With potential PPE supply disruptions and increased PPE needs in mind, PPE use by staff had to be judicious yet adequate enough to confer protection.”

Early research into the health and wellbeing of HCWs during the COVID-19 pandemic has linked access to adequate PPE with better psychological outcomes. Gold (2020) notes that their findings highlight the adverse effects that lack of PPE also have on mental health [2]. They add that insufficient PPE provision can be seen as institutional betrayal, described as “when trusted and powerful institutions act in ways that can harm those dependent on them for safety and wellbeing”, compounding trauma [2].

Another aspect of the multi-level coordination challenge, seen through the lens of PPE during the COVID-19 pandemic, is effective evidence translation and the challenges associated with rapidly changing national, regional, and organizational guidelines. Healthcare governing bodies in several countries including China, UK and USA altered official guidelines through 2020, impacting guidelines at regional and organizational levels [41–43]. In the USA, the Centers for Disease Control and Prevention (CDC) changed guidance on the use of N95 respirators on 11^th^ March 2020, outlining that HCWs could use a facemask where N95 respirators were not available. This guidance was contrary to previous CDC guidance that outlined the need for all HCWs to wear N95 respirators [43]. Similarly, in the UK, guidelines surrounding different aspects of PPE changed several times between March and April 2020 [42].

In our research, several respondents noted confusion around the correct PPE equipment for different areas of the hospital and for different staff. One respondent explained that staff within the organization were outright distrustful of organizational PPE guidelines, accusing the organization of trying to save money. This example outlines a challenge in knowledge translation in healthcare, but also the importance of trust in the organization and health system. In implementation science, the involvement of stakeholders (e.g., patients, providers, payers) in the design and introduction of initiatives is now seen as the ‘holy grail’ of healthcare improvement. However, such methods, including integrated knowledge translation, have not yet been well validated [44]. As such, tools to facilitate knowledge translation in this context will require greater attention to the understanding and matching of appropriate communication methods relevant for different stakeholders and audiences. Several tools developed by Knowledge Translation Canada’s Knowledge Translation Program, for example, can offer organizations guidance on communicating complex and simple information [45]. In the context of the COVID-19 pandemic and potential future pandemics where evidence generation and the need for knowledge translation moves at a particularly fast pace, healthcare organizations will benefit from having knowledge translation strategies in place ahead of time.

Similarly, effective staff engagement can aid knowledge translation and the build-up of trust between organization and staff, encouraging greater utilization of initiatives to improve HCWs health and wellbeing. Multiple respondents noted the importance of staff engagement in facilitating new initiatives, one noted.

“Our collective wisdom, at all levels of the organization, is huge. In giving voice to this, we not only find innovative and creative solutions, we also value and engage our workforce.”

The importance of staff input, feedback, and engagement across all levels of the organization was discussed frequently by participants who felt strongly that engagement between senior level managers and other staff had a two-fold value. As well as allowing the dissemination of the latest findings and COVID-19 guidelines, this engagement also offered staff the opportunity to raise ideas and concerns at the highest level, with the hope of making them feel valued and listened to.

### Mental health, stressors, and the role of fear

The prominence of mental health initiatives mentioned by the respondent group was somewhat unprecedented, given the infectious nature of the virus and the physical repercussions. However, it is possible that the wording of the case study questions, which requested information on either/both physical and mental health initiatives, encouraged participants to discuss mental health initiatives specifically. It may also point to an increasing awareness among the global health community of the far-reaching mental health implications of working and living through a global pandemic.

The role of fear as a barrier to the implementation of health and wellbeing initiatives for HCWs was a recurring theme among participants. They noted fear in the context of personal exposure, exposing family members to the virus should they transmit COVID-19 in their homes. One participant explained.

“Especially earlier on in the realization of the pandemic, [the] majority of the healthcare workers in my facility were fearful and concerned about their personal safety and the safety of their families. They didn’t trust that the organization had their interest at heart every day that they went to work and took care of patients (regardless of whether the patients were positive for COVID-19).”

Fear posed a particular challenge to the implementation of initiatives to adapt the healthcare facility to reduce transmission, as many participants noted that staff were hesitant to volunteer. Heads of Department were also hesitant to volunteer their staff for redeployment to higher demand services and units. Similarly, fear was noted as a challenge in duty rostering during the pandemic period as staff were concerned about undertaking higher risk activities. However, participants noted that such challenges were overcome through direct engagement with departments and staff, where concerns and fears were addressed, and with better training and assurance from peer groups.

In the pandemic situation, burnout is a real and tangible risk of increased pressure on healthcare services and on the health workforce. This is exacerbated due to the infectious nature of the disease, which reduces the capacity of the health workforce due to illness. Burnout is described as a “response to prolonged exposure to occupational stressors”, which may have serious consequences for healthcare professionals and the organizations in which they work [46]. Burnout is associated with sleep deprivation, medical errors, poor quality and safety of care, and low ratings of patient satisfaction [46]. Several of the respondents in the study reported burnout among multiple professional groups since the onset of the COVID-19 pandemic, with one suggesting that initiatives targeting HCWs health and wellbeing may struggle to reach those who need it most as a result of a lack of time and willingness to engage with the support on offer.

The importance of engaging with HCWs who are under extreme stress and pressure in a pandemic may pose a particular challenge, but it is nonetheless important to encourage uptake of mental health initiatives designed to improve their health and wellbeing. One participant noted that.

“Attention to emotional and mental well-being along with psychological support from immediate senior management and peer groups, managed to boost up the morale amongst the junior doctors. Continuous monitoring of the health and well-being of the staff in COVID-19 unit was done. Monitoring of the workload demands, personnel health and safety, resource needs and safe documentation practices was done.”

Such an example outlines that the range of actions and initiatives that must be employed simultaneously to ensure the mental health of HCWs is a critical consideration, while also considering how the very conditions that may be causing stress and burnout (e.g., workload demands) can be reduced to improve take up of additional initiatives. A consideration of these two elements together creates a positive cycle, where initiatives to reduce the stress burden on HCWs also free up time and energy for HCWs to better engage with the additional support on offer to improve mental health and wellbeing.

### Challenging the impact of misinformation

Conflicting information, misinformation and disinformation during the COVID-19 pandemic has been a novel challenge given it is the first pandemic in history in which technology and social media are being used on a massive scale as a means of keeping people connected and informed [47]. Respondents in this study largely highlighted both misinformation and disinformation as major challenges to facilitating initiatives for HCWs health and wellbeing, but some also noted the role of conflicting information in challenging implementation. One explained.

“The spread of misinformation via social media presented challenges to the implementation of both physical and psychological categories of welfare measures for staff, not just for the practice of IPC measures.”

Such is the importance of tackling misinformation and disinformation to aid the COVID-19 response globally, WHO Member States passed Resolution WHA73.1 at the World Health Assembly in May 2020 [48]. The Resolution recognizes that managing the infodemic is a critical part of controlling the COVID-19 pandemic: it calls on Member States to provide reliable COVID-19 content, take measures to counter mis- and disinformation and leverage digital technologies across the response. The Resolution also calls on international organizations to address mis- and disinformation in the digital sphere, work to prevent harmful cyber activities undermining the health response and support the provision of science-based data to the public [47, 48]. So too must health organizations consider the role that misinformation and disinformation may have in their COVID-19 response and on the health and wellbeing of their staff. One participant in the study noted that.

“Effective communication between senior staff/ leaders and staff is one way to address this issue. This involves timely dissemination of accurate and evidence-based information to staff, frequent engagement of staff by leaders to allay fears and address concerns, and two-way communication to ensure staff have avenues to provide feedback to leaders.”

Once again, addressing mis- and disinformation requires multi-level collaboration within healthcare organizations, clear preplanning, and engaging staff while respecting their ideas and thoughts. The provision of education and training for staff may also offer healthcare organizations the opportunity to counter mis- and dis-information with targeted scientifically-backed information on the origins, nature and symptoms of the virus, transmission and preventing transmission. This would benefit from including information on essential IPC within the healthcare setting, the role of testing, including available testing facilities for staff, and other common misconceptions. Providing clear information on where staff can find out more reliable information, speak to a dedicated helpline, or seek additional assistance within the organization also offers the opportunity to address mis- and dis-information on an ongoing basis. As the role of technology in day-to-day life and in healthcare continues to expand, more time must be invested in ensuring staff are able to access up-to-date and trusted information about the virus, the pandemic, and the national and local pandemic response.

### Developing new ways of working

The COVID-19 pandemic has shown HCWs and patients, their families, and carers the power of data and digital technology in tracking and containing the virus, and in developing new adapted ways of delivering healthcare [49]. There are a range of examples of telehealth being introduced for primary care in countries around the world, offering greater flexibility for patients and better reaching those in geographically challenging areas [50–52]. Similarly, in-person/telemedicine hybrid approaches to critical care have also been shown to be feasible and effective in addressing cross-cultural public health emergencies [53]. At the organizational level, several of our study participants developed new ways of working through the course of the pandemic. One participant explained.

“We had to close some of our clinics because of the pandemic of course, but then [had] to really think about how [we could] still serve our patients and encourage them to seek care if they need it. We had to do a lot of telemedicine, you know, on video, which worked really well, but that took a while to put the infrastructure in place.”

Changes to ways of working were largely designed to reduce the risk of transmission and optimize workflow given the increased pressure on resources. However, the development of new ways of working need not stop as the pandemic winds down. One respondent noted.

“As contingency spaces and capacities are gradually used to support the growth and development of the hospital, periodic reviews and re-investment efforts are critical to re-establishing such buffers. This would help to ensure that the hospital retains the capability and capacity to cope with future crises.”

It is notable that a lack of resources was a commonly highlighted barrier by participants in this research. Developing new, more efficient ways of working offers the opportunity for healthcare leadership to maximize the available resources. Of course, these advances must be closely monitored and evaluated to ensure standards are maintained or surpassed, the health and wellbeing of both patients and HCWs remain a priority, and that patient safety is a core consideration in any actions towards more efficient ways of working.

The COVID-19 pandemic has provided healthcare organizations around the world the opportunity to assess the present state of their ways of working, including the provisions on offer that seek to improve the health and wellbeing of their HCWs. As health systems around the world continue to address the pandemic, with an eye towards post-pandemic health system preparedness and planning, these considerations must remain at the heart of healthcare delivery and development.

### Limitations

The findings of the research offer insights into the facilitators and barriers to implementation only at one point in time. Findings therefore do not account for experiences of implementation after December 2020 and do not offer information on whether facilitators and barriers changed with time after initiatives were first introduced, nor whether additional facilitators and barriers have emerged in implementing new initiatives post-2020. However, the research offers valuable insight into facilitators and barriers in the beginning of the COVID-19 pandemic across a range of contexts that may be valuable through the course of the COVID-19 pandemic and for future pandemics and other prolonged crises. A further limitation of the study is the representativeness of the cases outlined. While the authors aimed to collect case studies from a range of geographic regions and types of healthcare organization (*N* = 13), the case study approach may have led to selection bias and so it is important to note that the findings are not necessarily representative of the experience of all healthcare organizations of that type/geography. Case study research has sometimes been criticized for lacking scientific precision in which to make a generalisation [11]. Nonetheless, the collective case study better facilitates studying multiple cases simultaneously to generate a broader appreciation of a particular issue [11]. As such, the research team designed the research to collect case studies and information from a range of organizations and health systems around the world to better assess trends ahead of generalization, while being cognizant of the limitations in representativeness of the case studies.

## Conclusions

HCWs at the frontline of the COVID-19 pandemic are at a disproportionate risk of adverse physical and psychological outcomes and so protecting HCWs requires a comprehensive and multi-modal approach to address multiple aspects of health and wellbeing. Through a case study approach, we demonstrate the facilitators and barriers to implementing such initiatives across healthcare organizations globally. Our findings, based on the experiences of 13 healthcare organizations, show multi-level coordination and preparedness is a critical starting point to ensure initiatives for HCW health and wellbeing can be implemented in a conducive environment, but it remains vital that the role of fear and misinformation must also be managed as the pandemic progresses. Health systems and healthcare organizations should now consider these findings at the system and organizational level as part of their efforts to design and implement smart and agile solutions for the physical and mental wellbeing of HCWs. Stakeholders must also recognize that the health and wellbeing needs of HCWs will continue well beyond the ‘end’ of the pandemic due to the prolonged impact of their experiences.

## Data Availability

Not applicable.

## Abbreviations

COVID-19: Severe acute respiratory syndrome coronavirus (SARS-CoV-2)
CDC: Centers for Disease Control and Prevention
IPC: Infection prevention and control
HCWs: Healthcare workers
LHSN: Leading Health Systems Network
LMICs: Low- and middle-income countries
PPE: Personal protective equipment
PSTRC: NIHR Imperial Patient Safety Translational Research Centre
WHO: World Health Organization
